# Gaucher-Associated Parkinsonism

**DOI:** 10.1007/s10571-015-0176-8

**Published:** 2015-03-29

**Authors:** Yaqiong Li, Ping Li, Huimin Liang, Zhiquan Zhao, Makoto Hashimoto, Jianshe Wei

**Affiliations:** 1 Laboratory of Brain Function and Disease, Institute for Brain Sciences Research, School of Life Sciences, Henan University, Kaifeng, 475004 China; 2 Tokyo Metropolitan Institute of Medical Sciences, 2-1-6 Kamikitazawa, Setagaya-ku, Tokyo, 156-0057 Japan

**Keywords:** Gaucher disease, Parkinsonism, Glucocerebrosidase, Synuclein, Lysosome

## Abstract

Gaucher disease is associated with Parkinson’s disease (PD) by mutations in glucocerebrosidase (GCase). The gene encoding GCase, glucosidase beta acid (*GBA*), is an important risk factor for PD. Findings from large studies have shown that patients with PD have an increased frequency of mutations in *GBA* and that *GBA* mutation carriers exhibit diverse parkinsonian phenotypes and Lewy body pathology. Although the mechanism for this association remains elusive, some hypotheses have been proposed to explain it, including gain of function caused by *GBA* mutations, which increases α-synuclein (α-syn) aggregation, loss of function due to lysosomal enzyme deficiency, which affects α-syn clearance, and even a bidirectional feedback loop, but each of these hypotheses has its limitations. It is also worth noting that many findings have implicated the interaction between α-syn and GCase, indicating the essential role of the interaction in the pathogenesis of *GBA*-associated parkinsonism. Therefore, the current review focuses on α-syn and GCase, and it provides some new thoughts that may be helpful for understanding the α-syn-GCase interaction and unraveling the exact mechanism underlying *GBA*-associated parkinsonism.

## Introduction

Gaucher disease (GD) is the most common lysosomal storage disorder and is caused by a deficiency in the enzyme glucocerebrosidase (GCase). It is an autosomal recessive disorder that primarily affects the mononuclear phagocyte system by lipid accumulation (Tayebi et al. 2003; Grabowski 2008). Patients typically manifest hepatosplenomegaly, hematological changes, anemia and orthopedic complications (Grabowski 2008; Choy and Campbell 2011). GD is classified into three types, on the basis of the presence or absence of neurological involvement: type 1, the most common form, has no associated neurological symptoms; type 2, or acute neuronopathic disease, displays severe neurological involvement leading to death within the first years of life; type 3, or chronic neuronopathic GD, exhibits varying degrees of systemic involvement with at least one neurological manifestation (Hruska et al. 2006; Siebert et al. 2014).

The gene encoding GCase, *GBA*, is located on chromosome 1q21–22 and comprises 11 exons encoding a 497 amino acid protein. So far, about 300 mutations of this gene have been identified, including frame-shift, point and splice site mutations as well as deletions, insertions and recombinant alleles (Choy et al. 2007; Hruska et al. 2008; Choy and Campbell 2011). Regarding the phenotype, many of these mutations have been classified as “null,” “mild” or “severe,” depending on the resulting level of GCase production. Null mutations, such as c.84dupG (84GG), do not lead to any enzyme production. Mild mutations, such as c.1226A>G (N370S), are only associated with type 1 disease. Severe mutations, such as c.1448T>C (L444P), result in enzyme production and are usually associated with type 2 or 3 disease (Beutler et al. 2005; Campbell and Choy 2012). Findings from more studies have shown that GBA is an important risk factor for Parkinson’s disease (PD). A small subset of GD patients with *GBA* carriers developed parkinsonian symptoms including tremor, rigidity and bradykinesia (Bembi et al. 2003) and were also associated with diffuse Lewy body (LB) pathology in the brain (Nishioka et al. 2011), with α-synuclein (α-syn) as the main component. This article reviews the present knowledge on the interaction of synuclein and GCase, with an emphasis on the pathogenesis and mechanism of *GBA*-associated parkinsonism.

## Gaucher Disease with Parkinsonism and Parkinson’s Disease in *GBA* Carriers

Parkinson’s disease is the second most common neurodegenerative disorder, affecting 1 % of people over the age of 65 worldwide (DePaolo et al. 2009). It is generally diagnosed after the age of 60 and causes motor dysfunctions, such as bradykinesia, resting tremor, rigidity and postural instability, but also affects autonomic functions and cognition (Poewe 2008). The two pathological features of PD are the death of neurons in certain regions of the brain and accumulation of proteins and lipids into structures. The former leads to the clinical picture of tremor, stiffness, slowness and difficulties with posture, and the latter generates LBs inside surviving neurons (Gai et al. 2000; Wei et al. 2009). LBs are composed predominantly of insoluble aggregates of the presynaptic protein α-syn. These neuropathologic aggregates are also associated with other neurodegenerative disorders including dementia with LBs (DLB) and multiple system atrophy (MSA) (Dev et al. 2003; Uversky and Eliezer 2009).

The pathogenesis of PD is unknown, although age and neurotoxins are associated risk factors. In recent decades, several causative genes have been identified, including three missense mutations, *A30P* (Krüger et al. 1998), *E46K* (Zarranz et al. 2004) and *A53T* (Polymeropoulos et al. 1997) of α-syn, which is strongly associated with early-onset PD (Singleton et al. 2003; Chartier-Harlin et al. 2004). Susceptibility studies suggest that contributing factors to PD include abnormal handling of misfolded proteins by the ubiquitin-proteasome system (UPS) and autophagy-lysosomal pathway (ALP), increased oxidative stress, and mitochondrial, lysosomal and other pathogenic dysfunctions (Lesage and Brice 2009).

Multiple independent studies have reported the association between *GBA* mutations and parkinsonism with an increased frequency of heterozygous *GBA* mutations in various cohorts of patients with parkinsonism (Westbroek et al. 2011). Furthermore, *GBA* mutation carriers exhibit parkinsonian phenotypes and present a diffuse pattern of LB distribution in the cerebral cortex (do Rosário Almeida 2012). The initial finding was that certain patients with GD exhibited parkinsonian symptoms and LB in the clinic, and additional reports were subsequently published (Bembi et al. 2003; Singleton et al. 2003; Chartier-Harlin et al. 2004; Westbroek et al. 2011). Mutations in the *GBA* gene were identified as from 4 to 28 % in cases with PD and LB disorders (Goker-Alpan et al. 2010; Clark et al. 2005). In 2003, a group was assembled of 17 such individuals of various ethnicities, including Ashkenazi Jewish patients. The patients in this series had relatively mild Gaucher manifestations with a mean age at diagnosis of 35 years (Tayebi et al. 2003). Their parkinsonian manifestations were similar to those noted in sporadic Parkinson’s disease, although many had an age of onset in their 40s, and most were younger than that for sporadic PD, although not all responded favorably to levodopa. These individuals exhibited asymmetric tremor, rigidity, akinesia and, at times, dementia, showing that cognitive changes had also occurred. Autopsies were carried out in the brain and showed LB inclusions, appearing GCase-positive on immunostains, with a more diffuse distribution involving in the cerebral cortex, hippocampus and brainstem/limbic regions (Nishioka et al. 2011; Neumann et al. 2009; Wong et al. 2004; do Rosário Almeida 2012). Taken with these studies, *GBA* mutation carriers have diverse LB pathologies in brain regions and phenotypes compared to sporadic PD patients.

It was noted that GD patients with parkinsonism frequently had relatives with parkinsonism who were heterozygous for *GBA* mutations (Goker-Alpan et al. 2004; Sidransky and Lopez 2012). In immunological analyses of brain tissue from GD patients and GD carriers with parkinsonism, all the samples with *GBA* mutations showed LB pathology (Shachar et al. 2011). Many other studies in different populations further support the link among GD, PD and LB disorders (Clark et al. 2005; Ziegler et al. 2007; Gan-Or et al. 2008; Mata et al. 2008; Kalinderi et al. 2009; Sidransky et al. 2009; Mitsui et al. 2009; Lesage et al. 2010; Mao et al. 2010). Recently, a strong association between PD and *GBA* mutations was demonstrated. A study of *GBA* in 57 brain bank samples from subjects with pathologically confirmed PD demonstrated that 12 % carried a mutation, which was significantly higher than the mutation frequency even in the at-risk Ashkenazi Jewish population (Lwin et al. 2004). Family studies revealed that among relatives of Gaucher probands, there was an increased number of individuals affected with parkinsonism (Goker-Alpan et al. 2004).

Importantly, several additional genetic studies in large patient cohorts demonstrated that patients with parkinsonism have an increased incidence of *GBA* mutations. In 2009, Sidransky et al. published a study that consisted of a collective analysis of 5691 patients with PD, complemented by 4898 controls, from 16 centers and across 12 countries. The full *GBA* coding region was screened, and loss-of-function mutations were observed in 6.9 % of cases and 1.3 % of controls. Among the Ashkenazi Jewish subset, higher mutation frequencies were seen: 19.3 % in cases and 4.1 % in controls (Sidransky et al. 2009; Bekris et al. 2010). Subsequent studies indicated an increased incidence of PD in relatives of GD patients, many of whom were carriers of *GBA* mutations (Goker-Alpan et al. 2004; Halperin et al. 2006), making *GBA* the most common known genetic risk factor for PD to date.

Furthermore, it is reported that non-motor symptoms (NMSs) are more frequent in PD patients with heterozygous glucocerebrosidase mutations (PD-GBA) (Brockmann et al. 2011). PD-GBA scores for abstraction and orientation were significantly lower. The overall NMS score was higher for PD-GBA than sporadic PD patients, and PD-GBA patients showed more severe cognitive dysfunction, apathy, depression and anxiety (McNeill et al. 2012a). Importantly, patients with GBA-positive PD had significantly less precise memory compared to cases with GBA-negative PD as well as GBA-positive individuals without PD (Zokaei et al. 2014).

Meanwhile, among GD patients and heterozygous carriers, hyposmia, cognitive dysfunction and parkinsonian motor signs are prevalent. They demonstrate olfaction and cognition impairments compared with controls. Hyposmia is the most common prodromal marker, 10 % in GD patients and GBA carriers, suggesting that hyposmia develops before cognitive dysfunction or motor abnormalities (McNeill et al. 2012b). Hyposmia also occurs in LBD, in which olfactory dysfunction in LBD and PD is associated with LB deposition in the olfactory bulb, nucleus and cortex (Williams et al. 2009; Ross et al. 2006). It proposes that hyposmia may be the most sensitive, but not a specific marker for identifying GD patients and carriers at greatest risk of developing PD.

## Proposed Mechanism for *GBA*-Associated Parkinsonism

The mechanisms underlying the relation between *GBA* mutations and the development of Parkinson’s disease remain elusive. However, recent studies provide some new perspectives. Generally, in autosomal recessive forms of Parkinson’s disease, such as those involving *PARK2*, *DJ*-*1* and *PINK1*, loss-of-function mutations are implicated, and these patients have an early onset of disease manifestation. By contrast, gain-of-function mutations, for example those in *SCNA* and *LRRK2,* are usually associated with autosomal dominant forms of Parkinson’s disease (Hardy et al. 2009; Sidransky and Lopez 2012). The recognition of the link among *GBA* mutants, GCase activity, SNCA and PD has made an important contribution to our thinking on the pathogenetic pathways in PD and dementia with LBs (Lwin et al. 2004; Sardi et al. 2011, 2013).

### Gain of Function

Support for gain-of-function mutations in *GBA* is mainly from the following findings. Both heterozygotes and homozygotes for GCase can develop PD. Most *GBA* mutations are missense mutations, which result in a misfolded protein. In keeping with this is the finding that mutant GCase is present within a significant proportion of α-syn inclusions in PD and GD brains (Sardi et al. 2011, 2013), suggesting that this abnormal conformation could contribute to the development of parkinsonism by increasing α-syn aggregation or directly interfering with the cellular autophagic-lysosomal mechanisms that mediate α-syn degradation (Cuervo et al. 2004). Furthermore, mutant GCase can accumulate in the endoplasmic reticulum, overwhelming the ubiquitin-proteasome pathway, burdening the lysosomal system or causing impairment of autophagy.

Some *GBA* mutations encountered in patients with parkinsonism are null mutations—a finding in conflict with the gain-of-function hypothesis. In fact, carriers of null alleles might have an even higher risk of developing parkinsonism (Neumann et al. 2009).

### Loss of Function

The loss-of-function theories mainly focus on altered lipid metabolism. The potential role of lipid homoeostasis in causing Parkinson’s disease is a topic of great interest. Parkinsonism could arise as a consequence of GCase deficiency because of the loss of function of the enzyme. Mutant protein leads to enzymatic deficiency and substrate accumulation. α-Syn can bind to the lipid raft-associated ganglioside GM1s (Martinez et al. 2007; Wei et al. 2009; Yap et al. 2013), so disordered glucosylceramide (GlcCer) metabolism is postulated to affect proper trafficking of α-syn to presynaptic membranes or to directly affect its fibrillization, leading to an accumulation of toxic α-syn species. Hence, lipid storage could cause changes in lipid homoeostasis, with subsequent alterations in synuclein processing (Fortin et al. 2004; Wei et al. 2009). In GCase KO mice markers of neurodegeneration, p62/SQSTM1, ubiquitinated proteins and insoluble α-synuclein accumulate in neurons and astrocytes lacking GBA. Mitochondria were dysfunctional and fragmented, with impaired respiration and reduced respiratory chain complex activities. Thus, a primary lysosomal defect causes accumulation of dysfunctional mitochondria as a result of impaired autophagy and dysfunctional proteasomal pathways (Osellame et al. 2013).

GCase enzyme activity was reduced in the substantia nigra and cerebellum in sporadic PD brains, and GCase deficiency was significantly observed in PD with GBA brain areas. For the most part, GCase protein expression was lower in PD with GBA and PD brains, as well also decreased in SH-SY5Y cells overexpressing α-syn or with PTEN-induced putative kinase 1 (PINK1) deficiency (Gegg et al. 2012). Similar results were observed in fibroblasts from patients with GD and heterozygous mutation carriers with and without Parkinson’s disease compared with controls (McNeill et al. 2014). This indicates increased α-syn levels or loss of mitochondrial function may cause GCase deficiency. The clinical finding that most patients with GD never develop Parkinson’s disease, despite low GCase activity, argues against a solely loss-of-function mechanism. This theory is supported by the low frequency of Parkinson’s disease reported in the large Gaucher cohort in the International Collaborative Gaucher Group (ICGG) Gaucher Registry (Rosenbloom et al. 2011). Thus, in most cases, the enzymatic deficiency cannot predict Parkinson’s disease.

### The Role of α-Synuclein in Parkinsonism with *GBA* Carriers

α-Syn, a kind of presynaptic protein, is widely recognized for its role in PD. Findings from recent studies have further suggested that α-syn is involved in the pathogenesis of *GBA*-associated parkinsonism. In the early stages of Parkinson’s disease, the GCase protein level and enzyme activity are selectively reduced. GCase deficits in sporadic Parkinson’s disease are related to the abnormal accumulation of α-syn (Murphy et al. 2014).

Recently, a selective interaction between α-syn and GCase was determined (Yap et al. 2011). The interaction occurred under lysosomal solution conditions (pH 5.5), and no significant interaction was noted at pH 7.4. Residue level mapping showed that the interaction site was at the α-syn C-terminal residues, 118–137. Immunoprecipitation and immunofluorescence studies verified this interaction in human tissue and neuronal cell culture, respectively. Intriguingly, the N370S mutant form of GCase has a reduced affinity for α-syn, so this binding at lysosomal pH might play an important role in facilitating α-syn degradation or preventing aggregation. Much of the processing of α-syn occurs in the cytoplasm, where this interaction was unexpected, but it offers a possibility for lysosomal pathways in α-syn degradation. When *GBA* is mutated or absent, the beneficial role of the interaction in α-syn processing is disrupted. These findings suggest that the α-syn-GCase association is favored in the lysosome and that understanding this noncovalent interaction provides the groundwork to explore molecular mechanisms linking PD with mutant *GBA* alleles.

Mazzulli et al. studied the association between α-syn pathology and GCase from a different perspective (Mazzulli et al. 2011). They performed a small hairpin RNA (shRNA)-mediated knockdown of GCase by lentiviral infection, which resulted in a 50 % reduction in GCase protein levels. Reduced concentrations of endoglycosidase H-resistant GCase were also reported, ascribed to the depletion of the lysosomal form of the enzyme. GCase knockdown increased the steady-state levels of α-syn 1.8-fold relative to controls, while the levels of tau, another disease-associated aggregation-prone protein, did not change. The same group then analyzed the dopaminergic neurons that were generated from induced pluripotent stem cells made from reprogrammed fibroblasts of a patient with GD. The results showed that these neurons also had an accumulation of α-syn that resulted in neurotoxicity attributed to aggregation-dependent mechanisms. By working with a mouse model and human brain samples, they concluded that intracellular GlcCer levels control the formation of soluble toxic α-syn oligomers and fibrils in cultured neurons, and mouse and human brain, leading to neurodegeneration. The promoted formation of α-syn assemblies further contributes to a pathogenic cycle by inhibiting the lysosomal maturation and activity of normal GCase, resulting in additional GlcCer accumulation and elevated α-syn oligomer formation.

However, studies of brain samples indicate that aggregation of α-syn is not related to GCase deficiency alone. Proteins extracted from cerebral cortex samples from patients with synucleinopathies with and without *GBA* mutations, as well as samples from control individuals and patients with GD, were studied by Western blotting. Patients with *GBA*-associated parkinsonism showed aggregated oligomeric forms of α-syn in the insoluble fraction, whereas only monomeric α-syn was noted in patients with *GBA* mutations without parkinsonism, including samples from patients with neuropathic GD (Choi et al. 2011).

Recent evidence in *Saccharomyces cerevisiae* and cell lines indicates that overexpression of α-syn has the ability to block endoplasmic reticulum-Golgi trafficking of proteins by inhibiting the formation of soluble *N*-ethylmaleimide-sensitive factor attachment protein receptor (SNARE) protein complexes (Cooper et al. 2006; Thayanidhi et al. 2010). Furthermore, Mazzulli et al. used primary cortical neurons to demonstrate that overexpression of either wild-type or A53T-mutant α-syn resulted in retention of GCase in the ER, associated with reduced lysosomal GCase activity (Mazzulli et al. 2011). They then confirmed this in vitro by studying GCase glycosylation patterns in PD and healthy control brain tissue. In addition, the same group analyzed human cortical material by GCase Western blot and showed that normal variation of α-syn protein levels modulates the lysosomal maturation and activity of GCase in vivo. Taken together, these data suggest that elevated levels of α-syn observed in PD and other synucleinopathies leads to decreased lysosomal activity of normal GCase (Mazzulli et al. 2011).

## Therapeutic Implication in α-syn and GCase Interaction

The proposed gain-of-function or loss-of-function mechanisms do not perfectly explain the association between PD and GD, and there are some clinical exceptions for each of them. However, α-syn is involved in the entire mechanism and plays an important role. Therefore, the focus should be on the altered function of α-syn in the observed association instead of a single gain or loss of function. A newly identified interaction between α-syn and GCase was reported that might influence cellular levels of α-syn by either promoting protein degradation and/or preventing aggregation (Sybertz and Krainc 2014; Yap et al. 2013). The authors also demonstrated that membrane-bound α-syn interacted with GCase and that this complex formation inhibited enzyme function. The interaction of α-syn and GCase in lysosomes may be beneficial to the degradation of α-syn, while intracellular GlcCer levels control the formation of soluble toxic α-syn oligomers and fibrils (Mazzulli et al. 2011; Sybertz and Krainc 2014; Yap et al. 2011). In other words, the interactions between α-syn and GCase and between α-syn and GlcCer have opposite effects on the aggregation of α-syn. Some *GBA* mutations result in misfolded GCase, which has no activity but can still interact with α-syn in lysosomes and contribute to the degradation. Thus, the aggregation of α-syn caused by GlcCer is compromised, which may explain why GD patients with low GCase activity will not develop parkinsonism. When *GBA* mutations affect the interaction site, like N370S, the beneficial role of the interaction in α-syn processing is disrupted, and the balance between aggregation and degradation is broken. This may be why carriers of null alleles might have an even higher risk of developing parkinsonism (Neumann et al. 2009). Furthermore, if the aggregation of α-syn cannot be cleared, it will further inhibit the lysosomal maturation and GCase activity, resulting in additional GlcCer accumulation and elevated α-syn. Recent data show that increasing GCase activity with adeno-associated viral vector (AAV) delivery of the enzyme into the brain of a GD mouse model can reduce the accumulation of SNCA, tau and ubiquitin (Sardi et al. 2013). This reduction was associated with an improvement in the memory defect. It suggested that increasing the glycosidase activity can modulate α-syn processing and may modulate the progression of α-synucleinopathies. In addition, neuronal glucosylceramide (GlcCer) accumulates to a certain threshold; a series of second events is triggered, which includes neuroinflammation and neurodegeneration, then subsequently causes neuronal death (Farfel-Becker et al. 2014). Hence, increasing the GCase activity in the CNS represents a potential therapeutic strategy for GBA-related and non-GBA-associated synucleinopathies, including PD (Schapira and Gegg 2013).

Since a reduction of glucosylceramidase protein levels and activity occurs in the brain of GD and PD patients with GBA mutation carriers, GCaes is a potential treatment target for GD therapy. Ambroxol hydrochloride, a small molecule, reduces alpha-synuclein levels in overexpressing neuroblastoma cells and markers of oxidative and endoplasmic reticulum stress in cells bearing GCase mutations. Ambroxol treatment results in a significant elevation of glucosylceramidase protein and activity levels in GD and PD with GBA mutations by activating the coordinated lysosomal expression and regulation network (McNeill et al. 2014). In addition, lysosomal activity of GC is tightly linked to expression of its trafficking receptor, the lysosomal integral membrane protein type-2 (LIMP-2), which acts as a chaperone helping to traffic glucosylceramidase from the endoplasmic reticulum to the lysosome; therefore, manipulating LIMP-2 expression to increase lysosomal GC activity is also a promising strategy for the treatment of synucleinopathies and GBA mutation carriers. Loss of GC activity leads to accumulation of its substrate glucosylceramide and α-synuclein. Heterologous expression of LIMP-2 accelerated clearance of overexpressed α-synuclein and promoted autophagic/lysosomal function, possibly by increasing lysosomal GC activity (Rothaug et al. 2014). Additionally, the receptor-interacting protein kinases (RIPKs), a class of serine/threonine protein kinases, are directly involved in the pathway of pathological events in severe forms of GD. The significant extension of lifespan and improvements of motor coordination in RIPK3 knockout mice support the notion that RIPK3 is not only a key activator of necrotic cell death, but also the RIPK3 pathway might be a molecular target for therapeutic intervention in GD (Vitner et al. 2014).

Further studies using techniques in cell biology, neuropathology and genetics are needed to better reveal the mechanisms that contribute to *GBA*-associated parkinsonism. The role of α-syn and GCase will continue to be a source of considerable interest in the field. Elucidating the factors underlying this association is necessary to improve genetic counseling for people who carry mutations in this gene. Moreover, a better understanding of the role of GCase in the development of parkinsonism will facilitate the development of new therapeutic strategies for *GBA*-associated parkinsonism.
